# Rural–Urban Suicide Mortality Disparities in High-Burden U.S. States: An Intersectional Analysis

**DOI:** 10.3390/healthcare14040533

**Published:** 2026-02-21

**Authors:** Bailey Smith, Kayli Marcum, Markisha Sowards, Cathryn Caudill, Meg Wright Sidle, Damian Cole

**Affiliations:** 1 Kentucky College of Osteopathic Medicine, University of Pikeville, Pikeville, KY 41501, USA; kaylimoore@upike.edu (K.M.); markishasowards@upike.edu (M.S.); 2 Department of Biomedical Sciences, Kentucky College of Osteopathic Medicine, University of Pikeville, Pikeville, KY 41501, USA; cathycaudill@upike.edu; 3 Institutional Research and Effectiveness, University of Pikeville, Pikeville, KY 41501, USA; msidle@upike.edu; 4 Department of Osteopathic Principles and Practices, Kentucky College of Osteopathic Medicine, University of Pikeville, Pikeville, KY 41501, USA; damiancole@upike.edu

**Keywords:** suicide mortality, rural health disparities, mental health inequities, rural—urban differences, intersectional analysis, American Indian and Alaska Native, public health epidemiology

## Abstract

**Background:** Suicide remains a leading cause of death in the United States, with more than 49,000 fatalities in 2023. Rural counties consistently face higher suicide mortality rates than urban areas, reflecting deep-seated mental health inequities. **Methods:** This study analyzes 39 U.S. states with suicide mortality rates exceeding the national average, as defined by the Centers for Disease Control and Prevention (CDC) (>14.1 per 100,000), to examine rural–urban disparities and their intersectional demographic factors. Age-adjusted mortality data (2019–2023) from HDPulse were analyzed using IBM SPSS Statistics, version 31.0. Counties were classified by USDA Rural–Urban Continuum Codes and stratified by region, sex, age, and race. Subgroup differences were tested using a two-way ANOVA (*p* < 0.01). **Results:** Rural suicide rates were significantly higher than urban rates (28.69 vs. 20.20 per 100,000; *p* < 0.001). The West reported the highest mortality and widest rural–urban gap (38.23 vs. 24.83), while the Northeast had the lowest. Men had higher rates than women, particularly in rural settings (37.12 vs. 11.77). The largest rural–urban gap occurred among young adults (20–39 years). American Indian/Alaska Native populations experienced the highest rates (rural: 58.73; urban: 35.15). The literature review highlighted limited healthcare access, social stigma, substance use, and economic hardship as variables commonly associated with rural–urban differences in suicide mortality. **Conclusions:** Suicide mortality is markedly elevated in rural America across all subgroups, with the greatest risks among young adults, men, and American Indian/Alaska Native populations. Tailored prevention strategies and expanded mental health infrastructure are critical for high-burden states.

## 1. Introduction

Suicide remains one of the most urgent public health challenges in the United States, consistently ranking among the leading causes of mortality [1]. In 2023 alone, over 49,000 Americans lost their lives to suicide—equivalent to one death every eleven minutes [1]. In the same year, 39 states had a suicide mortality rate greater than that of the national average (>14.1 per 100,000), underscoring the severity of the national crisis and the need for targeted intervention [2]. While suicide affects communities across the nation, its burden is disproportionately higher in rural areas, where rates have accelerated over the past two decades compared with urban counterparts [1].

Beyond rural–urban geography, suicide risk intersects with region, race, gender, and age in complex ways [3]. For example, states in the Western region of the United States consistently report the highest rates of suicide in the country [4]. Non-Hispanic American Indian and Alaska Native populations face suicide rates nearly double the national average, with a nearly 20% increase between 2015 and 2020 [5]. Gender differences add further complexity: men account for approximately 80% of suicide deaths, while women report higher rates of ideation and attempts [3,6]. Age is another crucial dimension, with suicide ranking as the second leading cause of death among individuals ages 10–34, a trend that has escalated alarmingly in recent years [7].

Multiple structural and environmental factors have been proposed to explain persistent rural–urban suicide disparities. Limited access to primary and behavioral-health services, provider shortages, and long travel distances can delay diagnosis and treatment of depression and substance-use disorders in rural populations. Rural residents also experience higher burdens of chronic medical conditions, including cardiovascular disease, chronic pain, and disability, which are associated with increased psychological distress and suicide risk. In addition, pervasive mental-health stigma and lower mental-health literacy in rural communities may discourage help-seeking and reduce early detection of psychiatric illness. Substance use—particularly opioid misuse—has also been identified as a critical contributor to suicide vulnerability in rural America. These overlapping structural, behavioral, and sociocultural factors highlight the need for intersectional and geographically informed analyses of suicide mortality [3,5].

Unlike prior studies that evaluate rural–urban disparities within single states or individual demographic groups, this study integrates a 39-state high-burden sample. The primary objective was to determine whether suicide mortality rates are significantly higher in rural compared with urban counties within states exceeding the 2023 national average. Secondary objectives were to assess whether the magnitude of rural–urban disparities varies by region, race/ethnicity, sex, and age through interaction modeling using independent t-tests and two-way ANOVA analyses. We hypothesized that suicide mortality rates would be significantly higher in rural counties and that this disparity would be amplified among specific demographic subgroups and geographic regions. Beyond quantifying disparities, the study further contextualizes findings through a targeted literature review to examine structural and environmental factors—including healthcare access limitations, chronic disease burden, mental-health stigma, substance-use patterns, and economic vulnerability—that have been described in association with persistent rural–urban differences. Through this multi-dimensional approach, the study seeks to describe the magnitude and contextual patterns associated with suicide disparities in high-burden states and to support future equity-oriented prevention planning.

## 2. Materials and Methods

**Figure 1:**
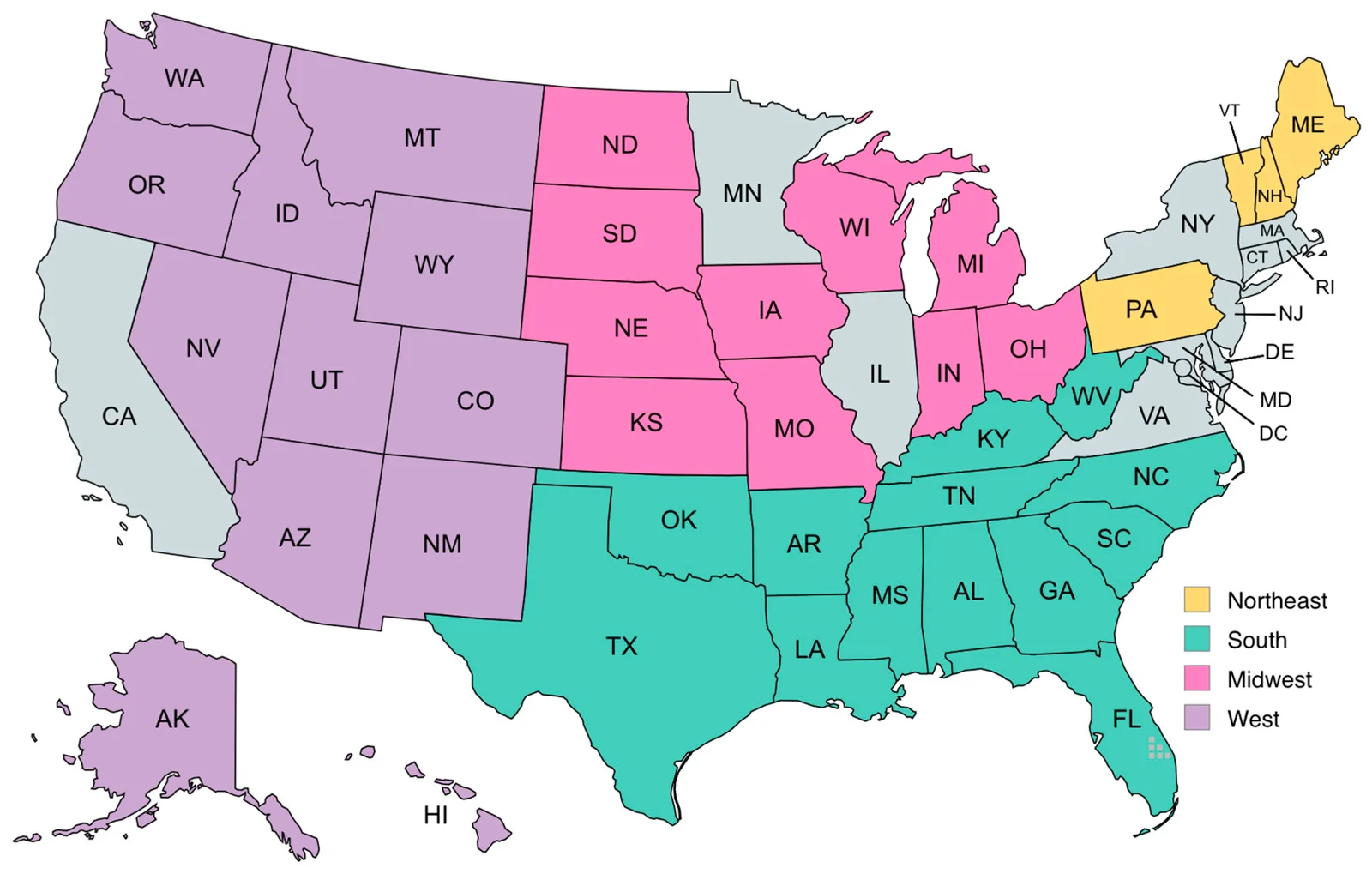
Geographic distribution of analyzed U.S. states by Census region. Map illustrating the states included in this study, organized according to U.S. Census Bureau regional classifications (Northeast, South, Midwest, and West). This visual representation provides geographic context for the regional comparisons conducted in the analysis and highlights the spatial distribution of high-burden states examined. The map was generated using MapChart (https://www.mapchart.net) and modified by the authors.

This study employed a cross-sectional design using secondary analysis of publicly available county-level mortality and population data to compare age-adjusted suicide and self-inflicted injury mortality rates between rural and urban counties. States were selected based on the Centers for Disease Control and Prevention (CDC) state-level mortality data indicating suicide rates in 2023 that exceeded the national average of 14.1 deaths per 100,000 population [2,4]. Using this criterion, as seen in Figure 1, 39 states were identified for inclusion in the analysis [2,4]. Restricting the sample to high-burden states allowed the analysis to focus on regions with elevated suicide mortality, where rural–urban differences may have important public health implications.

The states analyzed were organized by U.S. Census region as follows:

- Northeast: Maine, New Hampshire, Pennsylvania, and Vermont.
- South: Alabama, Arkansas, Florida, Georgia, Kentucky, Louisiana, Mississippi, North Carolina, Oklahoma, South Carolina, Tennessee, Texas, and West Virginia.
- Midwest: Indiana, Iowa, Kansas, Michigan, Missouri, Nebraska, North Dakota, Ohio, South Dakota, and Wisconsin.
- West: Alaska, Arizona, Colorado, Hawaii, Idaho, Montana, Nevada, New Mexico, Oregon, Utah, Washington, and Wyoming.

Mortality and population data were obtained from the NIH HDPulse database, which integrates Rural–Urban Continuum Codes (RUCC) developed by the United States Department of Agriculture (USDA) and U.S. population data files from the National Cancer Institute (NCI). Mortality and population data were obtained from the publicly available National Institute on Minority Health and Health Disparities (NIMHD) HDPulse database and Centers for Disease Control and Prevention (CDC) mortality data files at https://hdpulse.nimhd.nih.gov and https://wonder.cdc.gov. All data used in this study are publicly available, de-identified, and aggregated at the county level; therefore, institutional review board approval was not required. As the study involved secondary analysis of publicly available, de-identified data, informed consent from individual participants was not required.

There is no single, universally accepted definition of “rural” in the United States, which makes classification both challenging and context-dependent [8]. Federal agencies often rely on different criteria—ranging from population density and commuting flows to county size and metropolitan proximity—resulting in multiple, sometimes conflicting, ways to measure rurality [8]. This lack of uniformity can complicate research and policy comparisons. For this study, the classification system used by the NIH HDPulse database is utilized, employing the USDA’s Rural–Urban Continuum Codes (RUCC) [2]. The RUCC system categorizes counties along a nine-level scale that accounts for population size, urbanization, and adjacency to metropolitan areas [2]. In this framework, urban counties are generally larger and closely integrated with metropolitan economies, while rural counties tend to be smaller, more geographically isolated, and less connected to urban centers [2]. Although the USDA Rural–Urban Continuum Code (RUCC) system does not differentiate rural counties that functionally approximate metropolitan areas and does not fully capture within-county heterogeneity, it provides a consistent, nationally recognized classification framework that aligns with the NIH HDPulse database and allows for standardized rural–urban comparisons across states. The RUCC classification distinguishes counties based on population density and proximity to metropolitan areas, allowing for standardized differentiation between rural and urban regions. Counties with RUCCs 1–3 were classified as urban and codes 4–9 as rural, consistent with HDPulse classification. Death rates (per 100,000 population per year) were age-adjusted to the 2000 U.S. standard population and calculated using SEER*Stat software (Version 9.0.42.2, National Cancer Institute (NCI), USA), with population denominators based on U.S. Census counts as modified by NCI.

The suicide and self-inflicted injury mortality category included deaths corresponding to the following ICD-10 codes: U03 (Terrorism—suicide), X60–X84 (Intentional self-harm), and Y87.0 (Sequelae of intentional self-harm). Data suppression rules were applied in accordance with HDPulse confidentiality standards: data were not available (NA) or suppressed when fewer than 16 records were reported in a specific area–sex–race category to ensure confidentiality and stability of rate estimates. In instances where an average count of three was displayed, this denoted that the total number of deaths for the time period was 16 or greater (exceeding the suppression threshold) but rounded to three for privacy protection. Sample sizes and means may vary across demographic subgroups due to this suppression and rounding protocols.

To assess overall differences in suicide mortality between rural and urban counties, an independent samples t-test was conducted comparing mean age-adjusted rates across all counties. Subgroup analyses were performed by region (Northeast, South, Midwest, West), sex (male, female), age group (20–39, 40–64, ≥65 years), and race/ethnicity (White, Black or African American, American Indian or Alaska Native, Asian, Native Hawaiian or Pacific Islander, and Hispanic or Latino of any race). To evaluate differences across groups, two-way analysis of variance (ANOVA) models was applied, with county type (rural vs. urban) as the primary predictor. Separate models were constructed for region, sex, age group, and race/ethnicity. Each model included county type and the relevant subgroup variable. Statistical significance was set at *p* < 0.01 to minimize type I error. All analyses were conducted using IBM SPSS Statistics, version 31.0.

## 3. A Regional Sociodemographic Context of the 39 U.S. States

**Figure 2:**
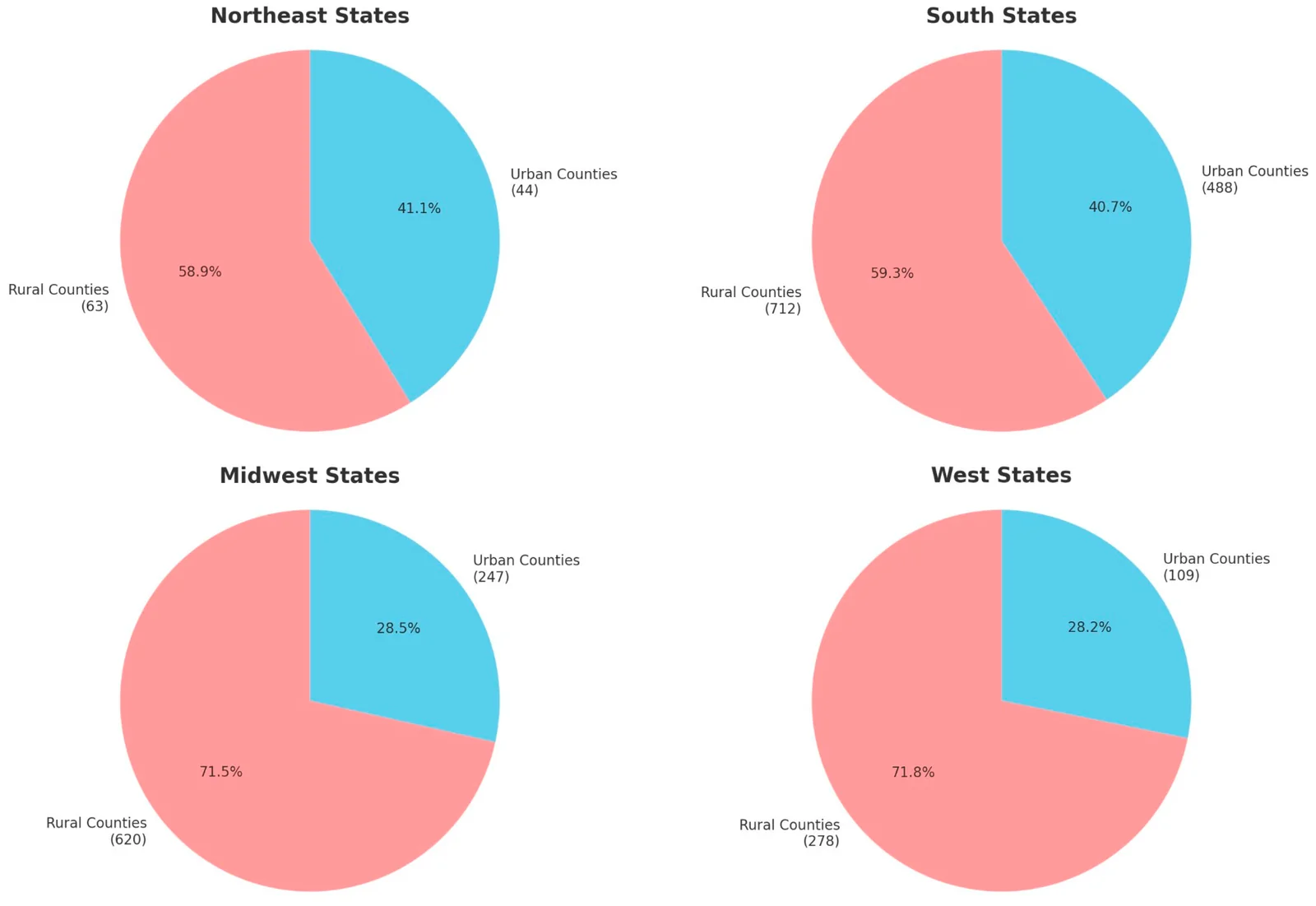
Regional Distribution of Rural vs. Urban Counties in the United States. This figure displays the proportion of rural versus urban counties across four U.S. Census regions among the 39 states included in this study [2]. Rural–urban county designation was derived using USDA Rural–Urban Continuum Codes, and county totals for each state were obtained from the NIH HDPulse database (reflect 2019–2023 data) [2]. Total county counts are displayed within each segment to enhance interpretation and highlight where rurality is most geographically concentrated.

To contextualize the sociodemographic heterogeneity across the regions analyzed, baseline geographic and socioeconomic characteristics were examined for 39 states using data from the NIH HDPulse database spanning 2019–2023. As shown in Figure 2, the geographic distribution of rural counties varies substantially across regions. Although the Northeast and South contain a high proportion of rural counties (58.9% and 59.3%, respectively), the Midwest and West exhibit markedly greater rural concentrations, with more than 71% of counties classified as rural.

**Figure 3:**
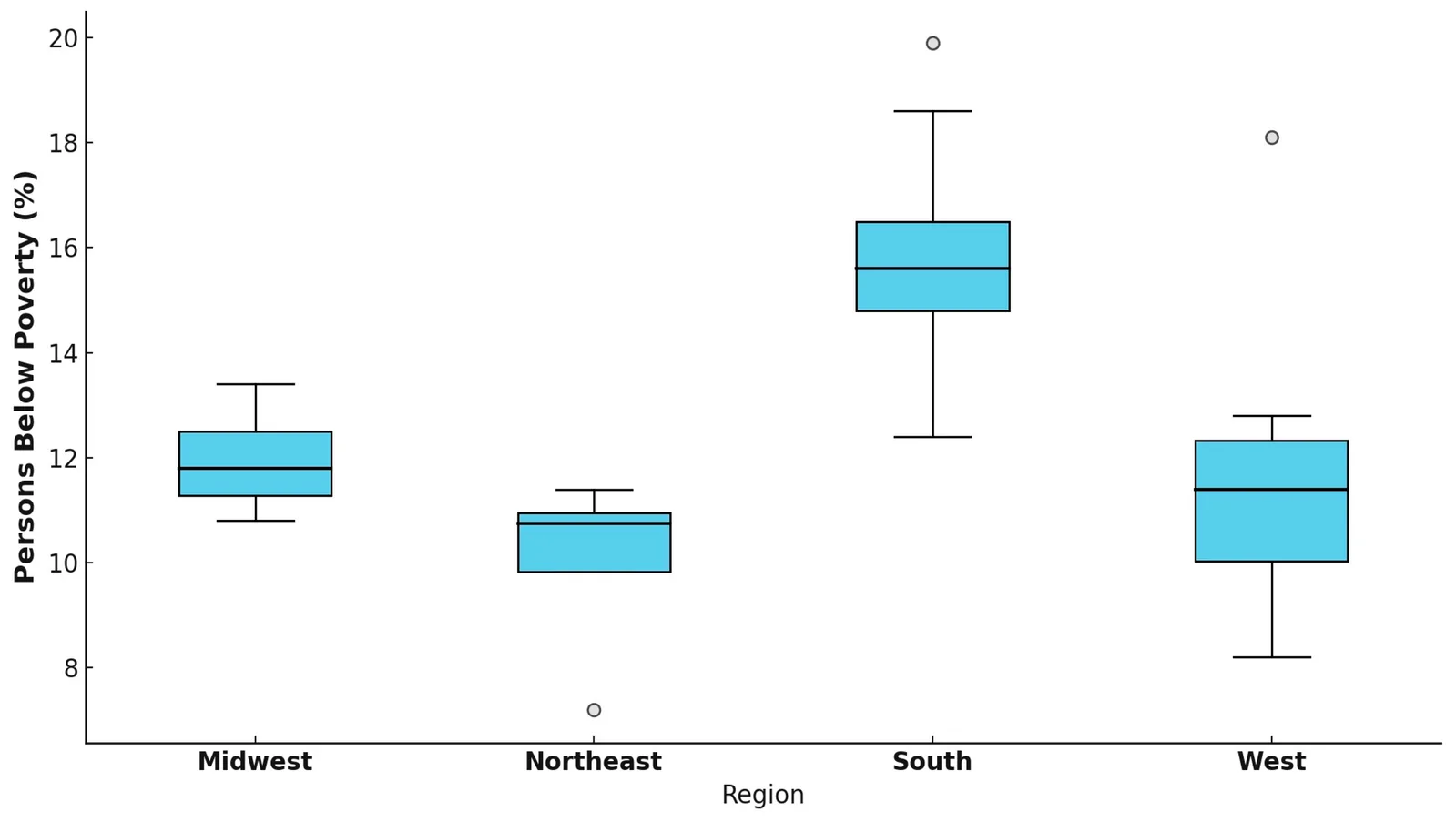
Distribution of Persons Below Poverty by U.S. Census Region (2019–2023). This figure illustrates the distribution of poverty rates among the 39 U.S. states included in the analysis, grouped into four U.S. Census regions: Northeast, South, Midwest, and West. Each box represents the interquartile range (IQR) of the percentage of persons below the federal poverty threshold between 2019 and 2023, with horizontal lines denoting median values and whiskers indicating minimum and maximum observations. All data were obtained from the NIH HDPulse database and reflect 2019–2023 statewide estimates [2].

**Figure 4:**
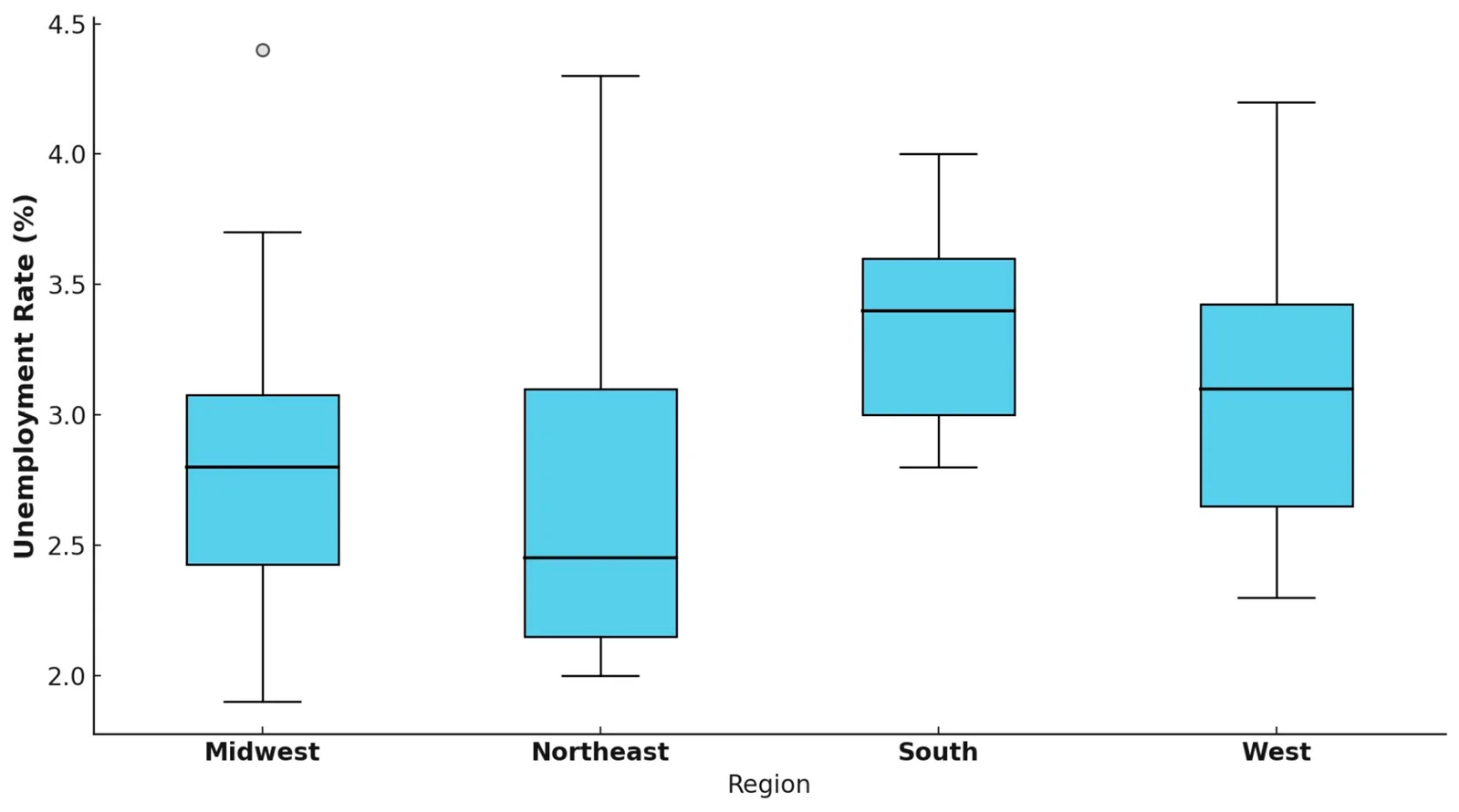
Distribution of Statewide Unemployment Rates by U.S. Census Region (2019–2023). This figure illustrates the distribution of unemployment rates among the 39 U.S. states included in the analysis, grouped into four U.S. Census regions: Northeast, South, Midwest, and West. Each box represents the interquartile range (IQR) of state-level unemployment rates between 2019 and 2023, with horizontal lines indicating median values and whiskers extending to the minimum and maximum observations. All unemployment data were obtained from the NIH HDPulse database and reflect 2019–2023 statewide estimates [2].

These geographic patterns are accompanied by pronounced regional socioeconomic disparities. The South demonstrates the highest mean poverty rate and the greatest intraregional variability, whereas the Northeast and Midwest show comparatively lower and more uniform poverty distributions (Figure 3). Economic vulnerability in the South is further reflected in Figure 4, which indicates both the highest median unemployment rates and the widest variability across counties, in contrast to the Northeast, which exhibits more stable employment patterns.

**Figure 5:**
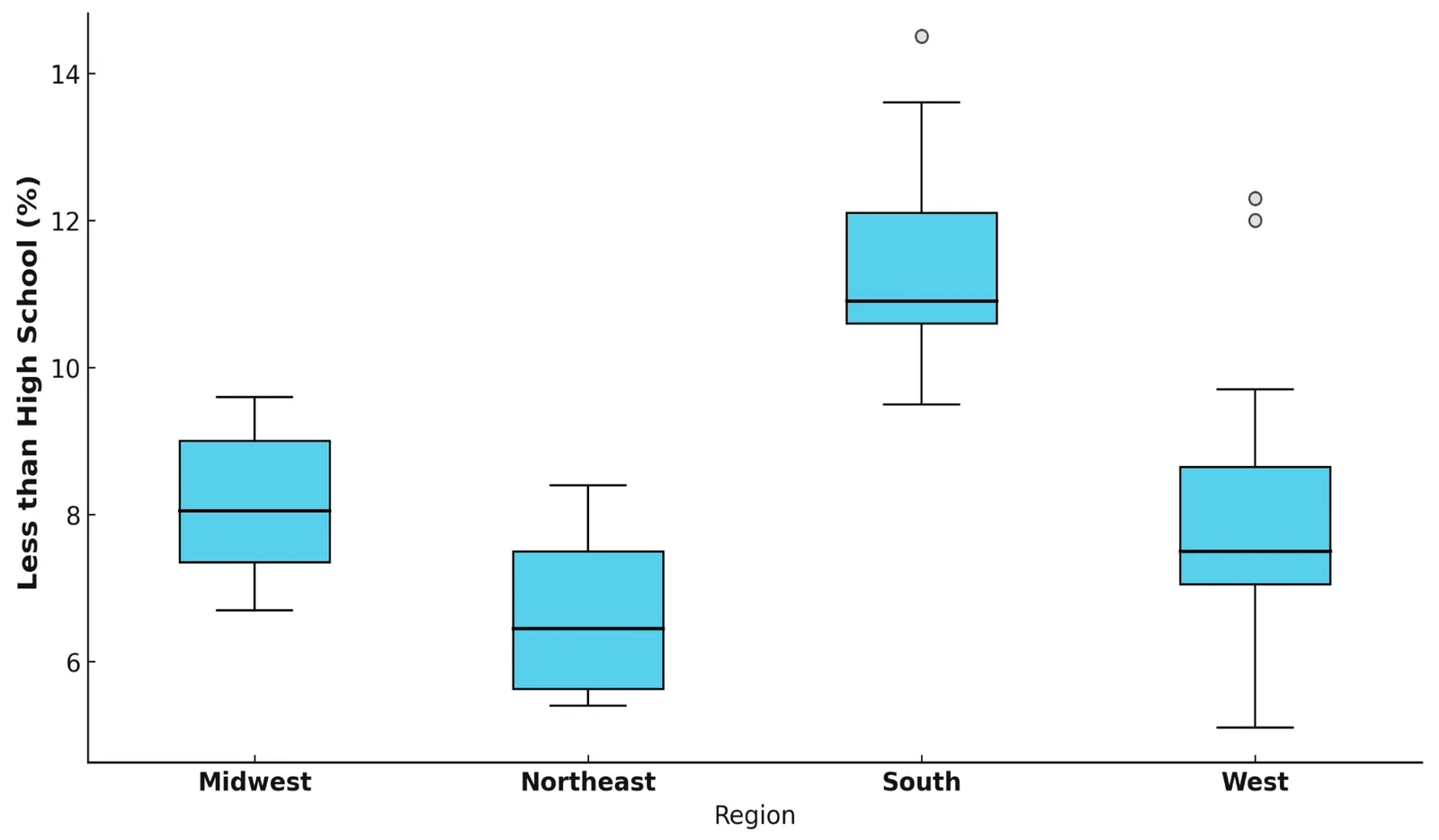
Distribution of Adults with Less than a High School Education by U.S. Census Region (2019–2023). This figure illustrates the distribution of adults aged 25 years and older with less than a high school education across 39 U.S. states, grouped by four U.S. Census regions: Northeast, South, Midwest, and West. Each box represents the interquartile range (IQR) of state-level proportions between 2019 and 2023, with median values indicated by horizontal lines. All education data were obtained from the NIH HDPulse database and reflect 2019–2023 statewide estimates [2].

**Figure 6:**
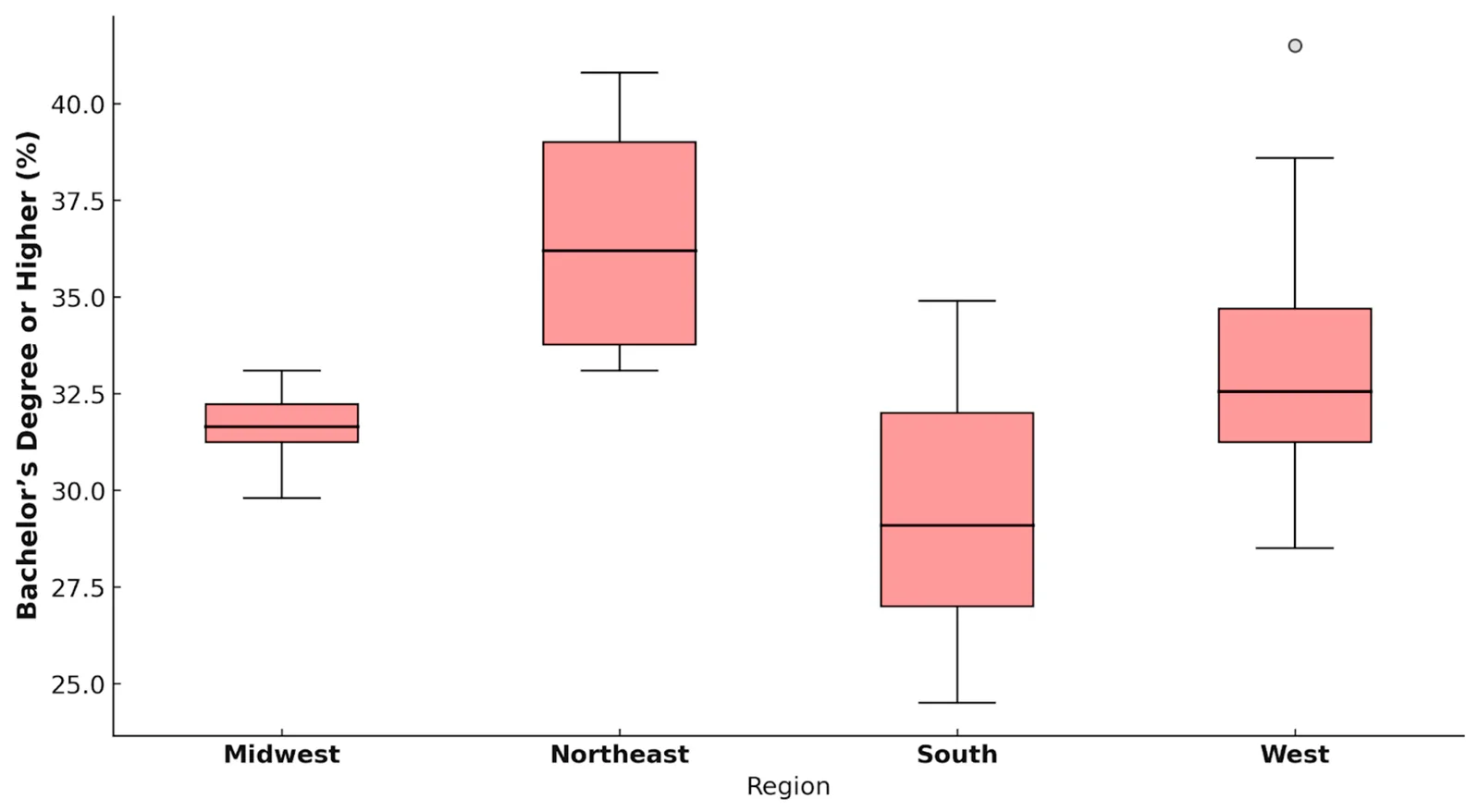
Distribution of Adults with Bachelor’s Degree or Higher by U.S. Census Region (2019–2023). This figure depicts the distribution of adults aged 25 years and older with at least a bachelor’s degree across 39 U.S. states, grouped by four U.S. Census regions: Northeast, South, Midwest, and West. Each box shows the interquartile range (IQR) of state-level educational attainment between 2019 and 2023. All education data were obtained from the NIH HDPulse database and reflect 2019–2023 statewide estimates [2].

Educational attainment also varies significantly by region. As illustrated in Figure 5, the South has the highest median proportion of adults without a high-school diploma. Conversely, Figure 6 shows that the Northeast and West exhibit the highest median levels of bachelor’s degree attainment with minimal variability, whereas the South displays both the lowest median attainment and the widest distribution, highlighting substantial regional inequities in access to higher education. Collectively, Figure 2, Figure 3, Figure 4, Figure 5 and Figure 6 illustrate pronounced interregional disparities in rurality and socioeconomic vulnerability across the United States, providing essential context for subsequent analyses.

## 4. Results

### Identifying High-Risk Rural Suicide Groups Through Intersectional Analysis of the 39 U.S. States

Analysis of the 39 high-burden states demonstrated a persistent rural–urban disparity in suicide mortality (Table 1). From 2019 to 2023, rural counties exhibited higher age-adjusted suicide rates than urban counties, with mean rates of 28.69 and 20.20 per 100,000, respectively (*p* < 0.001). Although urban areas recorded higher absolute annual counts, reflecting greater population density, mortality rates consistently indicated a rural disadvantage across all states. In accordance with HDPulse confidentiality standards, cells with fewer than 16 deaths within a specific area–sex–race category were suppressed to protect privacy and ensure statistical stability. Suppressed values were displayed using an imputed average count of three for reporting consistency and do not indicate low observed mortality within those groups.

Examination of demographic associations indicated that biological sex was the strongest predictor of suicide mortality, accounting for 55% of the explained variance. As shown in Table 2, men exhibited significantly higher suicide rates than women in both rural (37.12 vs. 11.77 per 100,000) and urban (29.17 vs. 7.16 per 100,000) counties (*p* < 0.0001). Although the interaction between sex and county type did not meet the predefined 1% significance threshold (*p* = 0.029), male predominance in suicide mortality remained consistent across geographic settings.

Age was also a significant determinant of suicide risk, with rural–urban disparities observed across all age strata (*p* < 0.001), as shown in Table 3. The largest rural–urban differential occurred among young adults aged 20–39 years, with a gap approaching 15 deaths per 100,000. Post hoc comparisons (Table 4) indicated significant differences across most age groups; however, suicide rates among middle-aged adults (40–64 years) and older adults (≥65 years) were not statistically different (*p* > 0.01). Overall, the model explained 15.6% of the variance in suicide mortality (R^2^ = 0.156), indicating a moderate joint effect of age and rurality on suicide risk.

Regional stratification further underscored this geographic gradient (Table 4). The West exhibited the highest overall mortality (31.34 per 100,000) and the widest rural–urban gap, with rates of 38.23 in rural counties versus 24.83 in urban counties, corresponding to an excess of 13.4 deaths per 100,000 in rural western communities. In contrast, the Northeast demonstrated the lowest overall mortality and the narrowest rural–urban differential. Although rural counties in the South and Midwest showed comparable rates, both regions exhibited a statistically significant rural disadvantage (*p* < 0.001), accounting for approximately 18% of the variance in mortality (R^2^ = 0.178).

The intersection of race/ethnicity and population density revealed substantial inequities in suicide mortality, as shown in Table 5, with the racial/ethnic model explaining 53% of the variance. American Indian/Alaska Native (AI/AN) populations experienced the highest suicide rates in both rural (58.73 per 100,000) and urban (35.15 per 100,000) counties, exceeding rates observed in all other racial/ethnic groups. White populations exhibited the second-highest mortality rates, whereas differences among Black, Hispanic, and Asian populations were not statistically significant (*p* > 0.01). These findings highlight a pronounced intersectional dimension to the rural suicide crisis, with the greatest burden concentrated among young adults, men, and AI/AN populations residing in rural areas.

## 5. Rural–Urban Sociodemographic Composition

### 5.1. Rural vs. Urban County Race and Age Profiles

Age and race profiles in rural and urban U.S. counties exhibit notable differences, which may influence patterns of suicide risk [9,10,11]. Rural populations in the U.S. tend to be older: for instance, between 2012 and 2016, 17.5% of rural Americans were aged 65 or older compared to 13.8% in urban areas [11]. More recent estimates suggest rural areas continue to “gray”: in 2023, the number of rural residents aged 65+ rose to 9.7 million, up from 7.4 million in 2010 [12]. In 2022, approximately 20% of the rural population was aged 65+ compared with 16% in urban counties [13]. Meanwhile, median age comparisons show rural America’s median age is around 43 years, versus 36 years in urban areas, reflecting fewer younger adults and a more aged resident base in rural counties [14]. Over time, rural counties have seen slower growth in younger cohorts and relative decline in prime working-age groups, partly driven by migration of young adults to urban areas [15].

In terms of racial and ethnic composition, rural areas are less diverse than their urban counterparts, though that gap is narrowing [16]. As of 2018, non-Hispanic White individuals comprised 78.2% of the rural population versus 57.3% in urban areas; Black Americans made up 7.8% of rural vs. 13.1% of urban populations [16]. Hispanics were 8.6% of rural populations, contrasted with 19.8% in urban areas [16]. Notably, American Indians and Alaska Natives represent a slightly higher proportion in rural locales (2.1%) than in urban regions (0.4%) [16]. However, rural America has become increasingly diverse, with racial and ethnic minority populations accounting for approximately 24% of rural residents in 2020 [17]. This evolving demographic landscape suggests that while rural America remains disproportionately White and older, the generational shift toward greater diversity may reshape future patterns of vulnerability [16,17].

### 5.2. Rural vs. Urban County Poverty Profiles

Poverty is a critical determinant of mental health disparities between rural and urban populations [18,19,20,21]. Poverty rates are consistently higher in rural communities, where financial hardship often limits access to health care and worsens mental health outcomes [19,20]. Because mental health screening is frequently integrated into primary care visits, individuals in rural areas who are uninsured or unable to afford routine medical care face substantial barriers to early detection and treatment of mental illness [21,22]. In urban settings, poverty is also prevalent; however, residents generally encounter fewer barriers to health care access [23]. Urban communities often benefit from government-funded health initiatives, expanded Medicaid programs, and a greater availability of low- or no-cost mental health services [24,25]. In addition, urban areas typically offer more employment opportunities, higher wage potential, and stronger social support infrastructures, all of which may mitigate some of the consequences of poverty [26,27]. By contrast, rural residents frequently lack comparable safety nets [28,29]. Lower population density often results in fewer government and nonprofit initiatives, while generational poverty persists due to limited educational and economic opportunities [28,29]. These structural disadvantages, compounded by social isolation and persistent stigma surrounding mental health treatment, position rural poverty as an associated factor for poor mental health outcomes [22,29,30]. In addition, concerns regarding confidentiality and perceived lack of anonymity in small rural communities may further discourage engagement in mental-health screening and treatment, reinforcing stigma-related barriers to early detection [21,29].

### 5.3. Rural vs. Urban County Unemployment

Unemployment has historically been more persistent and volatile in rural areas compared to urban labor markets [27,31]. Data from the U.S. Bureau of Labor Statistics show that during the early phase of the COVID-19 pandemic, unemployment spiked to 13.3% in urban areas and 11.4% in rural areas [32]. While metropolitan labor markets rebounded relatively quickly, many rural areas experienced prolonged recovery periods [32]. Even in non-crisis periods, the rural unemployment rate has tended to remain 0.5–1.0 percentage points higher than that of urban counties [33]. This disparity reflects structural vulnerabilities: rural counties are more reliant on a limited set of industries and often have lower levels of educational attainment and higher rates of out-migration among young workers [12,15,34]. Consequently, while recessions may initially hit urban labor markets harder, rural economies frequently endure longer durations of elevated unemployment, leaving persistent geographic gaps in access to stable employment opportunities [27,35].

In addition to higher unemployment, rural areas are characterized by a labor market composition that is more heavily weighted toward blue-collar employment (e.g., manual, trade, agricultural, or industrial work), which carries greater physical demands and occupational risks [12,27,36,37]. According to occupational data from the U.S. Bureau of Labor Statistics (BLS), production jobs account for 10.8% of rural employment compared with 5.7% in urban areas, and transportation and material-moving jobs are also disproportionately concentrated in rural regions [12]. USDA and Federal Reserve reports confirm that rural economies remain more reliant on agriculture, mining, and manufacturing, while urban areas concentrate in service-oriented and professional occupations [12,27,33,35]. These jobs—particularly in agriculture, construction, and mining—frequently involve heavy lifting, prolonged standing, awkward body postures, and routine exposure to hazardous machinery [12,27,36]. These physical demands are well documented in the U.S. Bureau of Labor Statistics (BLS) Occupational Requirements Surveys and National Institute for Occupational Safety and Health (NIOSH) injury-surveillance reports [38,39]. As a result, rural workers not only face greater barriers to stable employment but also disproportionately engage in physically strenuous and higher-risk occupations, compounding disparities in both economic security and health outcomes [27,36,37].

### 5.4. Rural vs. Urban County Education Attainment

Educational attainment in rural counties continues to lag behind that of urban areas, reflecting long-standing structural disparities with far-reaching consequences [12,27,31]. While adults in rural regions are more likely to hold a high school diploma as their highest level of education, residents of cities and suburban counties attain bachelor’s and advanced degrees at substantially higher rates [12,40]. From 2000 to 2023, the share of adults with a bachelor’s degree or higher rose in rural areas from approximately 15% to 23%, while urban areas experienced an even steeper increase of 26% to 38% in the same time span [41]. These gaps emerge early in the educational pipeline: rural students face unique barriers such as limited school resources, geographic isolation, and reduced access to postsecondary pathways, which collectively diminish their likelihood of earning a college degree compared to peers in more urbanized locales [12,15,27]. Such disparities restrict not only individual economic mobility but also the collective social and health capital of rural communities, perpetuating cycles of inequality that widen the divide between rural and urban populations [12,15,27,31].

## 6. Exploring Other Associated Factors of Rural–Urban Suicide Disparities in the U.S.

### 6.1. Rural vs. Urban County Access to Primary Care, Mental Health Resources and Chronic Health Conditions

Access to healthcare in rural America is constrained by persistent provider shortages and greater travel distances required to reach clinical services [9,12,21,25]. Only 12% of U.S. physicians practice in rural communities, despite 61% of federally designated health-professional shortage areas being rural [21]. Even when rural residents have a regular source of care, these providers are less likely to be physicians and are often located much farther from patients than providers in urban settings [42,43]. Limited access to both professional and digital health information further constrains rural patients’ ability to make informed care decisions [23]. These structural barriers are consistently associated with inequities in health outcomes observed among rural residents [21,22,25]. Reduced availability of behavioral health care—and the resulting delays in diagnosing and treating depression and substance-use disorders—further amplify vulnerability to mental-health crises in rural communities [9,21,25]. These factors combined help to explain why, in 2022, the suicide mortality was found to be nearly 50% higher in rural areas compared with urban counties (20.0 vs. 13.4 per 100,000) [44].

To compound these primary-care limitations, significant disparities also exist in the availability of mental-health facilities [25]. Rural counties are substantially less likely to host community-based treatment facilities—particularly those serving youth—and more than 60% of federally designated mental-health professional shortage areas are located in rural regions [22,25]. In 2020, approximately 65% of rural counties have no practicing psychiatrist even though about one-fifth of those living in rural areas, or about 6.5 million individuals, have a mental illness [22]. Evidence from a national county-level analysis published in JAMA Pediatrics found that each incremental shortage in the mental-health workforce was associated with a 4% increase in youth suicide rates [45]. These patterns mirror other national findings showing that mental-health shortage-area counties experience significantly higher suicide rates, with the effect most pronounced in rural regions [45].

Beyond shortages in healthcare and behavioral-health services, rural residents face elevated burdens of chronic illness, health-risk behaviors, and premature mortality—each associated with psychosocial stress and heightened vulnerability to suicide [12,13,18,21,22]. Rural Americans experience higher rates of death from the five leading causes of death—heart disease, cancer, unintentional injury, chronic lower respiratory disease, and stroke—compared with urban residents [46,47]. For example, during 2006–2015, rural counties experienced higher cancer mortality than urban counties [180 vs. 166 deaths per 100,000) [48]. Rural adults are more likely to smoke, be obese, and report lower levels of physical activity [49]. In addition, rural residents tend to be older and in poorer health, which contributes to higher rates of chronic diseases including cardiovascular disease [49]. By 2016, the rural–urban mortality gap had widened to 135 excess deaths per 100,000 persons [50]. These patterns underscore the cumulative effects of behavioral and chronic-disease burdens that intensify psychological distress and functional decline [21,29,30].

### 6.2. Addressing Limited Access to Primary Care and Mental Health Resources

Limited access to both primary care and behavioral-health services is consistently associated with rural–urban disparities in suicide mortality [21]. As this study demonstrated, suicide rates were significantly higher in rural counties—particularly in the West and South—where provider shortages and delayed access to care are most severe [9,10,25]. Expanding rural residency programs, strengthening loan-forgiveness initiatives, and increasing the number of Certified Community Behavioral Health Clinics (CCBHCs) in high-burden counties are evidence-based approaches that can help alleviate provider shortages and improve early intervention [21,22,25,51]. Integrating behavioral-health screening into primary-care and community-clinic settings may further enhance early detection and continuity of care, reducing fragmentation across service systems [21,22,25]. Moreover, expanding funding for Rural Health Clinics (RHCs) and Community Health Centers, deploying mobile health units in frontier areas, and embedding mental-health assessments within chronic-disease management visits could simultaneously address comorbid physical and psychological burdens [21,22,25]. Aligning chronic-disease management with mental-health screening is especially critical given that rural adults experience higher rates of cardiovascular disease, diabetes, and chronic pain—conditions strongly associated with depression and suicidal ideation [12,13,21,22]. These combined strategies can strengthen population resilience and help narrow the persistent rural–urban gap in suicide mortality [9,10,21,22].

Telehealth offers a transformative solution to mitigate these access disparities [52,53]. Robust evidence demonstrates that telepsychiatry and virtual cognitive-behavioral therapy yield outcomes comparable to in-person treatment for depression, anxiety, and suicidality [53]. In a national cohort of more than 16,000 U.S. veterans, each 1% increase in tele-mental-health utilization corresponded to a 2.5% reduction in suicide-related events, underscoring the measurable population-level benefits of virtual care [53,54]. For rural communities, where travel distances, and transportation barriers continue to limit in-person access, telehealth enables timely crisis intervention, ongoing follow-up, and continuity of care across geographic boundaries [21,25,29,53,54].

### 6.3. Rural vs. Urban County Stigma Influence on Mental Health

Stigma surrounding mental-health care remains one of the most pervasive barriers to treatment in rural America [21,29]. It not only discourages help-seeking but also deepens the social isolation already faced by these communities [9,21,29]. In sparsely populated areas with limited anonymity, individuals often fear being outcast for seeking psychological help, reinforcing silence and withdrawal rather than openness to care [9,21,29]. Older adults in isolated rural counties consistently report the highest levels of both public and self-stigma and exhibit lower psychological openness—factors that discourage discussion of mental distress even within trusted networks [22,55,56]. This silence perpetuates a self-reinforcing cycle: stigma fuels isolation, and isolation intensifies untreated psychological distress [21,29,55,56].

In contrast, urban counties—with denser provider networks and more normalized mental-health discourse—offer residents greater opportunities to disclose distress and access treatment without fear of community judgment [21,22,25,29]. Compounding these differences, rural, low-income populations face lower mental-health literacy, higher poverty, and limited insurance coverage, which intersect with stigma to further suppress help-seeking behavior [23,24,28,29]. Collectively, stigma functions as both a cultural and structural determinant of mental-health inequity [21,22,29].

### 6.4. Reducing Stigma and Improving Mental Health Literacy

Reducing stigma and improving mental-health literacy are essential for mitigating the rural–urban suicide mortality gap [10,21,22,23,29]. Rural residents experience higher levels of self-stigma and lower psychological openness than urban populations, making early intervention and community-based outreach especially important [21,29,55,56]. Anti-stigma efforts must therefore be localized, drawing upon trusted community institutions such as schools, churches, and cooperative extension programs to normalize help-seeking and increase engagement [21,22,29]. Implementing mental-health literacy initiatives—such as school-based mental-health curricula, and continuing-education modules for rural clinicians in stigma-sensitive communication—can foster earlier recognition of psychological distress [21,22,23,29]. These interventions should be culturally adapted to align with rural norms of self-reliance and strong community identity [21,22,29]. By increasing familiarity with mental-health concepts, improving perceived confidentiality, and building trust in local providers, such initiatives can break the cycle of stigma-induced isolation [21,22,23,29]. When paired with expanded care access and tele-mental-health integration, these approaches have the potential to restore early detection, strengthen community resilience, and ultimately reduce the excess suicide mortality documented in rural counties [9,10,21,53,54].

### 6.5. Substance Use and Firearms Role in Rural–Urban Suicide Disparities

Substance use, particularly opioid misuse, has been frequently examined in relation to suicide mortality patterns in rural America [13]. Rural residents experience a disproportionate burden of overdose events and substance-related harm: in one multi-state sample, 45.9% of rural participants reported a prior overdose compared with 31.6% of urban participants, and rural individuals experienced significantly more lifetime overdoses [57]. Although national hospitalization rates for substance-related conditions have declined in recent years, outcomes remain markedly worse for rural patients [58,59]. Rural patients hospitalized for substance-related conditions often experience more complex and challenging clinical courses, a pattern tied to the limited availability of hospital-based screening, medications for opioid use disorder, and addiction consult services in rural settings [58].

Compounding these risks are substantial geographic inequities in access to evidence-based addiction treatment [58]. Buprenorphine, a partial opioid agonist used in medication-assisted treatment (MAT) to reduce cravings and withdrawal symptoms, remains considerably less available in rural communities [58]. Nationwide, 26% of U.S. counties have fewer than four buprenorphine-wavered clinicians, and 7% have none [60]. As a result, large geographic regions—particularly sparsely populated states such as Alaska, Wyoming, and South Dakota—lack practical access to office-based buprenorphine treatment or opioid-treatment programs (OTPs) [60]. Long travel distances further reduce treatment retention; approximately 25% of methadone patients travel ≥15 miles for care, and 8% commute > 50 miles, which is associated with shorter treatment duration and poorer adherence [61]. These access gaps exist despite rising need: in the Appalachian region, rural counties consistently recorded the highest opioid-related mortality from 2018 to 2021, exceeding urban areas [62].

These intersecting patterns—higher overdose exposure, more severe clinical complications, and chronically limited access to medication-assisted treatment—help contextualize the elevated rural suicide mortality [63,64]. Geographic isolation and fragmented treatment access amplify existing social and economic vulnerabilities in rural counties, creating conditions in which substance-use disorders can progress unchecked and suicide risk escalates more rapidly than in urban settings [21,22].

Beyond substance use disorders and overlapping psychosocial vulnerabilities, rural–urban disparities in suicide mortality are strongly influenced by differential access to lethal means, particularly firearms [65,66,67]. Although firearm-related homicides occur more frequently in urban settings, suicides account for the majority of firearm deaths in rural counties, comprising up to 80% of all gun-related fatalities [65,67]. Notably, when firearm-related deaths are excluded, suicide mortality rates in rural regions decline markedly, underscoring the central role of firearm availability in rural suicide risk [65]. This association is particularly concerning given that firearm access is substantially greater in rural communities [65,66]. Among rural households with adolescents, approximately 90% report the presence of firearms; however, more than 60% of these firearms are stored unsecured and frequently loaded, significantly elevating the risk of suicide and other forms of self-inflicted injury [66].

### 6.6. Strengthening Substance Use Resources and Reducing Firearm Suicides

Reducing suicide risk in rural populations requires parallel investment in addiction treatment and crisis-response systems [22,51]. Although violent crime is more concentrated in urban centers, rural counties experience disproportionately high rates of opioid prescribing, overdose deaths, and untreated substance-use disorders—factors strongly linked to suicidality [19,57,62,64]. Expanding medication-assisted treatment (MAT) through additional OTPs and office-based buprenorphine programs is essential, especially in frontier states and those without Medicaid expansion, where access remains severely limited [24,58,60,61]. Increasing funding for recovery housing, transportation assistance, and mobile addiction-treatment units can further mitigate spatial barriers to care [21,58,60,61].

Building on this need for expanded treatment access, rural communities also require more effective crisis-response mechanisms [21,22,51]. Implementation of the Crisis Intervention Team (CIT) and co-response models—which pair officers with mental-health professionals—has shown promise in diverting individuals from incarceration toward treatment and reducing crisis-related fatalities [68,69]. These coordinated approaches recognize that suicide prevention in rural America extends beyond individual therapy: it demands interlinked behavioral-health, substance-use, and justice-system frameworks that can address trauma, addiction, and social instability simultaneously [21,22,51,57]. By expanding treatment accessibility, enhancing crisis-response capacity, and improving care coordination, such systems can help reverse the compounding cycle of substance misuse, isolation, and suicide that disproportionately affects rural communities [18,21,22,51].

To address the role of firearm access in shaping rural–urban suicide disparities, it is necessary to consider the temporal dynamics of suicidal crises and the lethality of readily available means. The majority of suicide attempts occur within one hour of the onset of suicidal ideation; in this context, the ready availability of firearms in rural settings—contrasted with administrative and regulatory barriers that contribute to lower firearm ownership rates in urban areas—can convert an otherwise transient psychological crisis into a fatal outcome at disproportionately high rates [65,67]. Consequently, reducing the rural–urban suicide gap requires not only expanded access to behavioral health services, but also targeted lethal-means safety interventions, including education on secure firearm storage, to introduce protective barriers during periods of both acute and chronic psychological distress [65,66].

### 6.7. Rural vs. Urban County Medicare and Medicaid Usage

Rural populations in the United States experience disproportionately greater health and financial burdens under both the Medicare and Medicaid systems, which play a central role in shaping behavioral-health access and suicide risk [19,21,22,24]. Medicaid, in particular, serves as a critical safety net for low-income and medically vulnerable rural residents, yet reimbursement limitations, provider shortages, and inconsistent state-level eligibility policies restrict access to mental-health and substance-use services [21,22,24]. Medicare-covered individuals in rural regions also have higher rates of chronic illness, contributing to elevated vulnerability to depression, functional decline, and suicide risk [11,18,21,70]. Despite providing essential coverage, rural Medicare beneficiaries—especially those enrolled in Medicare Advantage—report greater financial strain, narrower provider networks, and more limited mental-health benefits than their urban counterparts [70,71,72]. These combined constraints delay access to counseling, psychiatric evaluation, and follow-up care, thereby increasing vulnerability to crises such as major depressive episodes or suicidal ideation [18,21,22]. This pattern reflects higher rates of poverty, disability, and chronic illness in rural communities—all factors associated with increased suicide mortality [11,18,21,24].

### 6.8. Enhancing Medicare and Medicaid Support for Behavioral Health

Given rural Americans’ greater reliance on public insurance, Medicare and Medicaid policies represent important structural components of the broader healthcare landscape within which rural–urban suicide mortality disparities are observed [11,21,22,24]. Evidence from the CDC demonstrates that higher county-level insurance coverage is associated with lower suicide mortality, highlighting the importance of expanding public-insurance pathways in high-burden regions [42]. A key strategy is expanding the reimbursable behavioral-health workforce [25]. Equally important is ensuring reimbursement parity for tele-behavioral-health services within Medicare and Medicaid [73]. Telehealth has proven capacity to overcome the geographic isolation, transportation barriers, and clinician shortages that rural residents face, making sustained telehealth integration vital for continuity of care [21,22,25]. Finally, stabilizing the financial infrastructure of Critical Access Hospitals, Rural Health Clinics, and community mental-health centers is critical, as these facilities often represent the only behavioral-health resources available in rural counties [19,21,22,24]. Strengthening reimbursement and operational support across these domains may be relevant within broader efforts to address longstanding inequities in healthcare access observed in rural communities [19,21,22,24].

## 7. Discussion

### 7.1. Overview of Rural–Urban Suicide Mortality Disparities

This study provides a comprehensive multi-state analysis of rural–urban suicide mortality disparities across the 39 U.S. states whose 2023 suicide rates exceeded the national average. Using HDPulse mortality data (2019–2023) and two-way ANOVA models, we identified significant geographic, demographic, and socioeconomic patterns that highlight the structural and contextual factors associated with elevated rural suicide mortality.

### 7.2. Regional Variation in Rural–Urban Suicide Mortality

Consistent with prior surveillance, rural counties exhibited significantly higher suicide mortality than urban counties (*p* < 0.001). However, the magnitude of this gap varied substantially across regions, reaffirming the importance of geographic context. The West demonstrated the highest overall mortality and the largest rural–urban disparity (+13.4 deaths per 100,000), followed by the South and Midwest, while the Northeast showed the smallest gap. These findings align with broader regional patterns—including provider shortages, geographic isolation, and limited crisis-care infrastructure documented in the West and South—and highlight the importance of regionally tailored public health planning rather than uniform national approaches.

### 7.3. Sociodemographic and Intersectional Disparities

Demographic patterns further illustrated the intersectional nature of suicide disparities. Sex was a strong main effect (*p* < 0.001), with males consistently experiencing higher mortality across both rural and urban settings. Race and ethnicity showed the most pronounced interaction with geography: American Indian/Alaska Native and White populations in rural counties experienced the highest mortality, and the significant Race × County Type interaction (*p* < 0.001) indicated that rural context intensifies racial disparities. These findings are consistent with national data documenting elevated suicide mortality among AI/AN communities—often concentrated in remote areas with chronic underfunding of behavioral-health services—and among White working-class rural adults confronting economic instability, substance-use burden, and elevated firearm accessibility.

### 7.4. Suicide Disparities in American Indian and Alaska Native Populations

These findings highlight the importance of understanding the structural and contextual drivers underlying suicide disparities in American Indian and Alaska Native populations [74,75]. The disproportionately high suicide mortality observed among American Indian and Alaska Native populations likely reflects longstanding structural and systemic inequities in access to culturally appropriate and geographically accessible behavioral-health services [74,75]. Many AI/AN communities are located in rural or frontier regions, where mental-health infrastructure is limited and travel distances to care are substantial [74,75]. Although the Indian Health Service (IHS) plays a central role in delivering healthcare, chronic underfunding, workforce shortages, and variability in resources across regions continue to restrict timely access to behavioral-health and crisis services [74]. Historical trauma, intergenerational adversity, and persistent socioeconomic disadvantage further contribute to psychological distress and suicide vulnerability [74]. In addition, social determinants of health—including employment, education, housing stability, and community conditions—have been identified as key drivers of suicide risk in AI/AN populations [75]. Strengthening culturally grounded, community-led prevention strategies and expanding behavioral-health capacity within tribal and rural health systems are therefore essential to reducing these disparities [75].

### 7.5. Age and Life-Course Vulnerability

Age-related findings also revealed meaningful geographic variation. Although suicide rates were elevated across all age groups, disparities were greatest among adults aged 20–39 years in rural areas, who often face compounding risks: social isolation, financial strain, and limited access to primary and behavioral health care. The significant Age × County Type interaction (*p* < 0.001) underscores that suicide prevention efforts must account for differing life-course vulnerabilities. Expanding employer and Medicaid-based behavioral-health screening, strengthening rural primary-care integration, and embedding mental-health support into chronic-disease management could reduce risk across rural working-age and older adult populations. While rural population aging represents an important contextual factor, the cross-sectional design precludes assessment of temporal trends, and future longitudinal analyses are needed to evaluate how demographic shifts intersect with suicide mortality patterns over time.

### 7.6. Structural and Environmental Associated Factors

The contextual synthesis in this study situates these statistical patterns within broader structural and environmental characteristics of rural communities. These structural and environmental factors were not directly measured in the present study because the analysis relied on publicly available secondary mortality data. Instead, they are discussed to contextualize the observed disparities and highlight potential mechanisms supported by prior literature. Provider shortages, limited mental-health and primary-care infrastructure, delays in diagnosis and treatment, high burdens of chronic disease, reduced insurance coverage, and persistent stigma collectively constrain help-seeking and continuity of care. Substance-use patterns, especially opioid misuse, further interact with geographic isolation and treatment shortages to heighten vulnerability. These structural disadvantages align closely with the significant regional and demographic interactions observed in the statistical models. Although substance use and economic hardship are widely recognized correlates of suicide mortality, these variables were not directly modeled due to dataset constraints, underscoring the need for future analyses incorporating multilevel socioeconomic and behavioral-health indicators.

### 7.7. Public Health and Policy Implications

Taken together, the findings indicate that rural–urban suicide disparities are observed within broader structural differences across healthcare systems, labor markets, social environments, and public-insurance frameworks. Continued examination of behavioral-health infrastructure, telehealth expansion, workforce distribution, culturally tailored prevention efforts, and crisis-service integration will be important for understanding how these systems intersect with rural suicide mortality patterns.

### 7.8. Strengths and Contributions of the Study

A key contribution of this study is its focused examination of 39 U.S. states with suicide mortality exceeding the national average, representing a high-burden population that has received limited dedicated, multi-state intersectional analysis. Unlike prior studies that often examine rurality or demographic factors in isolation, this study explicitly models interaction effects using a two-way ANOVA framework to quantify how region, race, sex, and age modify the association between county type and suicide mortality. By integrating recent multi-year county-level mortality data with structural, sociodemographic, and health-system contextualization, this work provides a more geographically granular and analytically rigorous characterization of rural suicide disparities. This intersectional approach advances current understanding by identifying where disparities are most pronounced and which populations and regions are most likely to benefit from targeted, region-specific prevention strategies.

## 8. Limitations

Several limitations should be considered when interpreting this study’s findings. First, the analysis relied on secondary data sources (NIH HDPulse, CDC WONDER, USDA RUCC) from 2019 to 2023, which may include reporting delays, regional inconsistencies, and misclassification of suicide deaths, particularly in rural coroner-based systems, potentially producing conservative mortality estimates. County classification was based on the USDA Rural–Urban Continuum Codes, which, although widely used and consistent with the NIH HDPulse framework, may not fully capture the functional complexity of counties that border metropolitan areas or reflect heterogeneity within counties. In addition, HDPulse confidentiality rules require suppression or rounding of small cell counts when fewer than 16 deaths occur within specific area–sex–race strata, which may reduce sample sizes in certain demographic subgroups and limit the precision of subgroup-specific rate estimates. The study period (2019–2023) overlaps with the COVID-19 pandemic, which represents a potential temporal modifier of suicide mortality patterns. Pandemic-related factors (including social isolation, economic instability, substance-use fluctuations, disruptions in in-person behavioral-health services, and rapid telehealth expansion) may have differentially affected rural and urban communities. Because the present analysis relied on multi-year aggregated rates rather than year-specific trend modeling, it was not designed to isolate pandemic-specific temporal effects. As such, a portion of the observed rural–urban differences may reflect pandemic-associated temporal dynamics rather than stable structural disparities alone. Additionally, restricting the sample to states exceeding the 2023 national suicide mortality average (14.1 per 100,000) may limit generalizability, and findings could differ if alternative thresholds were applied or if all 50 states were included in the analysis.

Second, the cross-sectional ecological design identifies associations but cannot establish causality, and aggregate county-level measures may mask within-county disparities, including those affecting subpopulations such as veterans or AI/AN communities living in mixed rural–urban areas. Because the analysis relied on county-level aggregate data, findings reflect area-level associations and should not be interpreted as individual-level suicide risk estimates; substantial heterogeneity may exist within large rural counties that include micropolitan areas, towns, or tribal lands with distinct healthcare infrastructures and social contexts. Third, while the two-way ANOVA framework allowed assessment of key interactions (Region × County Type, Race × County Type, Age × County Type), model precision was limited by the granularity of available county-level covariates. Additionally, the use of regional means may attenuate extreme values in sparsely populated counties with unstable annual rates. Despite these limitations, the study’s large multi-state sample, robust two-way modeling strategy, and use of recent mortality data strengthen confidence in the observed patterns and reinforce the need for regionally tailored, community-based suicide-prevention strategies.

## 9. Conclusions

This study demonstrates that suicide mortality in the United States is shaped by a complex intersection of geography, demography, and socioeconomic context, with rural communities bearing a disproportionate burden. Across 39 high-burden states, rural counties consistently experienced significantly higher suicide mortality than urban counties, with the largest disparities concentrated in the West and South. These patterns highlight that structural and health-system inequities—rather than individual factors alone—are strongly associated with the excess suicide burden observed in rural America.

Demographic differences further reinforce the intersectional nature of suicide risk, particularly among males, younger adults (20–39 years), and American Indian/Alaska Native and White populations in rural regions. Together with the contextual findings on provider shortages, poverty, substance use, and limited public-insurance infrastructure, these results suggest that multiple layers of disadvantage converge to elevate suicide mortality in rural communities.

Reducing the rural–urban suicide gap will require sustained investment in behavioral-health capacity, expanded telehealth and broadband access, strengthened crisis-response systems, and targeted approaches that reflect the needs of specific regions and demographic groups. By integrating quantitative disparities with structural context, this study advances a multidimensional understanding of suicide risk and underscores the importance of regionally tailored, equity-focused prevention strategies.

Future research should incorporate longitudinal and multilevel modeling, include structural variables such as firearm availability and broadband access, and integrate community-engaged methods to capture lived experiences. Evaluating the long-term effects of telehealth expansion, workforce initiatives, and policy reforms will be essential to determining whether emerging interventions can meaningfully reduce rural suicide mortality. Ultimately, meaningful progress will depend on addressing the structural barriers that restrict access to care and perpetuate vulnerability in rural communities.

## Figures and Tables

**Table 1:** Rural vs. Urban Suicide Rates (All 39 States, 2019–2023).

Measure	Rural Counties (N = 3069)	Urban Counties (N = 3947)	Effect Size (Cohen’s *d*)	*p*-Value
Age-adjusted suicide rate (mean)	28.69	20.20	0.69 (medium)	<0.001
Average annual count (mean)	6.68	21.61	0.55 (medium)	<0.001

**Table 2:** Suicide Rates by Sex and County Type.

Sex	Rural Mean Rate	Urban Mean Rate	Sex Mean	Effect Size (Cohen’s *d*)	*p*-Value
Male	37.12	29.17	33.15	0.67 (medium)	<0.001
Female	11.77	7.16	7.72	1.00 (large)	<0.001
Interaction (Sex × County Type)	-	-	-	<0.1 (weak) Partial Eta^2^	0.029

**Table 3:** Suicide Rates by Age Group and County Type.

Age Group	Rural Mean Rate	Urban Mean Rate	Age Group Mean	Effect Size (Cohen’s *d*)	*p*-Value
20–39 years	38.15	23.58	27.74	0.67 (medium)	<0.001
40–64 years	31.18	22.92	25.27	0.87 (large)	<0.001
65+ years	32.73	21.61	23.66	1.11 (large)	<0.001
Interaction (Age Group × County Type)	-	-	-	0.01 (small) Partial Eta^2^	<0.001

**Table 4:** Suicide Rates by U.S. Region and County Type.

Region	Rural Mean	Urban Mean	Rural–Urban Difference	Regional Mean	Effect Size (Cohen’s *d*)	Significance
West	38.23	24.83	+13.40	31.34	0.70 (medium)	*p* < 0.001 (highest)
South	27.30	19.44	+7.86	22.56	0.81 (large)	n.s. vs. Midwest
Midwest	26.00	19.68	+6.32	22.62	0.61 (medium)	n.s. vs. South
Northeast	22.62	17.23	+5.39	20.29	0.80 (large)	*p* < 0.001 (lowest)
Interaction (Region × County Type)	-	-	-	-	0.01 (small) Partial Eta^2^	<0.001

**Table 5:** Suicide Rates by Race/Ethnicity and County Type.

Race/Ethnicity	Rural Mean Rate	Urban Mean Rate	Race/Ethnicity Mean	Effect Size (Cohen’s *d*)	*p*-Value
American Indian/Alaska Native	58.73	35.15	48.25	1.02 (large)	<0.001
White	24.19	NA	-	-	-
Hispanic or Latino	21.46	10.59	11.67	1.59 (large)	<0.001
Asian/Pacific Islander	15.15	8.86	9.09	3.32(large)	<0.001
Black	8.60	10.16	10.15	0.59(medium)	<0.001
Interaction (Race/Ethnicity × County Type)	-	-	-	0.02 (small) Partial Eta^2^	<0.001

Note: Urban White suicide rates were suppressed in the HDPulse database due to confidentiality and are therefore not reported.

## Data Availability

The data analyzed in this study were obtained from publicly available resources in the National Institute on Minority Health and Health Disparities (NIMHD) HDPulse database (https://hdpulse.nimhd.nih.gov) and were used for secondary data analysis. Derived datasets and statistical outputs generated during the current study are available from the corresponding author upon reasonable request.

## Abbreviations

The following abbreviations are used in this manuscript:

ANOVA	Analysis of Variance
BLS	Bureau of Labor Statistics
CCBHC	Certified Community Behavioral Health Clinic
CIT	Crisis Intervention Team
CDC	Centers of Disease Control
IQR	Interquartile Range
MAT	Medication Assisted Treatment
NIOSH	National Institute for Occupational Safety and Health
NCI	National Cancer Institute
OTP	Opioid Treatment Programs
RHC	Rural Health Clinics
RUCC	Rural–Urban Continuum Codes
USDA	United States Department of Agriculture
